# A Case

**Published:** 1892-11

**Authors:** Charles B. Atkinson

**Affiliations:** New York


### A CASE.[1]

BY CHARLES B. ATKINSON, D.D.S., NEW YORK.

A RECENT case has interesting features, although it is unfortunate that it is not uncommon in this day of great opportunity for the acquirement of general professional ability.

February 17, 1892, a female, eighteen years of age, of lymphatic temperament, several carious teeth, as noted later, chronic incontinence of urine as a sequel of scarlet fever, and a general hyperæsthetic condition of the mucous membrane, presented with considerable swelling over the left side of the face, especially prominent opposite the site of the left superior first molar, extending backward and downward to a point near the lower border of the inferior maxilla, posterior to the site of the left inferior second molar, and having a poultice of soap and sugar about the size of a silver dollar placed about the latter point. The skin was greatly inflamed under and about this poultice, and extremely thin, and threatening to slough through momentarily. On removing the poultice (which was at once ordered off permanently) a fistulous opening was disclosed, which discharged serous fluid. A probe demonstrated soft tissue in all directions, bleeding freely on slight pressure of the probe.

No abnormal growth of any kind was present. This swelling at the angle of the jaw on direct pressure produced a sensation of a hard growth at the angle, but traction on the skin anteriorly and posteriorly permitted a clear definition of the normal maxilla, and plainly disclosed the hardness to be confined to the soft tissue and entirely due to the inflammation about the fistula (*caused by the poultice*).

Examination of the interior of the mouth showed the absence of the superior centrals, carious left superior cuspid, first and second bicuspid, and first molar crown absent with its three roots separated, anterior buccal root very loose, great gingival hypertrophy, bleeding on slightest pressure, with extensive swelling on the buccal aspect of the gum back to the condyle. The left superior second molar carious, and left superior third molar also carious in the crown, erupting outwardly and posteriorly, and impinging on the ramus at each closure of the jaw to such an extent as to cause considerable pain.

---

[1] Read before the California State Dental Association, July, 1892.

The mucous membrane covering the ramus at this point was quite inflamed and considerably swelled.

The left inferior third molar was carious in the crown (or, as Dr. Kirk suggests, contained a "morsal" cavity). The left inferior first molar was absent, having been extracted (?) March, 1891. The left inferior second molar absent.

A small abscess pointed opposite the site of the posterior root of the left inferior second molar, on the buccal aspect of the inferior maxilla, with slight swelling.

Pressure about this point caused a slight exudate of this pus, and the finger being carried over the apparently healthy gum between the left inferior bicuspid and third molar demonstrated a sharp, pricking sensation, irregularly marked along the buccal border of the space from which the left inferior first and second molars had been removed.

*Diagnosis.*—Fractured buccal wall of the alveolus of the left inferior first molar, which had become carious.

Inferior external swelling sympathetic (poultice, wanton interference).

Superior external swelling due to the impinging of the left superior third molar (erupting) against the ramus. These swellings aggravated, and, in a measure, joined to produce, a pendulous enlargement of the left side of the face by the œdema from inflammation about the roots of the left superior first molar.

*Treatment.*—The poultice was discontinued at once, as stated.

The external swelling was coated with contractile collodion.

The loose anterior root of the left superior first molar was removed.

A U-bolt was fitted to the posterior buccal and palatal roots, and a porcelain crown set thereon, the hypertrophied gum having been thoroughly excised and burned with pure carbolic acid. The bite was slightly opened by filling with cement to form an abnormal crown on the left superior second molar.

A filling of cement was placed in the left superior third molar, so as to occlude upon an inclined plane with the posterior edge of the left inferior second molar, diverting the erupting superior third molar forwardly and inwardly, and prolonging the buccal face with a rounded, smooth surface, against which the ramus could play without inconvenience.

The gum between the left inferior second bicuspid and third molar was laid open, and the carious alveolus removed with a bone burr in the engine.

This line of treatment proceeded from February 17, 1892, until March 16, 1892, when the case had assumed a normal appearance and feeling: it is now being fitted with bridges and permanent fillings.

From the following history of this case, previous to its presentation to the writer, some grave conclusions may be drawn, and it was the special nature of its history which urged its presentation before you. In the writer's experience it is by no means an isolated example of hasty diagnosis with unfortunate results. In this instance a comely young woman sustains a cicatrix needlessly; although by constant anointing with lanolin and persistent manipulation the scar has already been drawn down under the border of the inferior maxilla (a distance of at least three-fourths of an inch from its original site), and although it will be small and not readily noticed, still it will be a life-long scar. In addition to this is the year (nearly) of needless physical and mental suffering, with the systemic exhaustion the continual urge to suppuration induced.

*History.*—The left inferior first molar was extracted (?) in the latter part of March, 1891, under nitrous oxide. In about two weeks the left side of the face began to swell. The family physician was called in, who said he feared an abscess, although he hoped it would not be of the bone. On further examination he said the left inferior second molar had abscessed, and advised extraction. The patient consulted a dentist, but her naturally flabby tissue had become so tense from the serous infiltration that the dentist could not gain access to the tooth. Another call in a week resulted likewise.

July 1 (three months after the first extraction) six teeth were removed, including the abscessed left inferior second molar.

July 4 the physician was again called, and diagnosticated the (sympathetic) external swelling as an abscess and ordered a flax-seed *poultice!*

This poultice was continued until August 1 (a month), when the swelling was lanced, and the contents proved to be chiefly blood. The poultice was continued for three weeks, and again the lance was plunged into the patient's cheek.

The flaxseed poultice was then replaced with one of soap and sugar. The hard swelling and the before-mentioned neighborhood enlargements continued. The patient visited the country in the last week of August, where she consulted a surgeon in a hospital, who diagnosticated a bone tumor, the traumatic removal of which would probably result fatally, and advised its removal by injection. This operation was declined.

From September, 1891, until February, 1892, the soap and sugar continued its fell work of softening and thinning the skin beneath the plaster, until the case presented to the writer, February 17, 1892, with the results stated.

